# Prion Infectivity and PrP^BSE^ in the Peripheral and Central Nervous System of Cattle 8 Months Post Oral BSE Challenge

**DOI:** 10.3390/ijms222111310

**Published:** 2021-10-20

**Authors:** Ivett Ackermann, Reiner Ulrich, Kerstin Tauscher, Olanrewaju I. Fatola, Markus Keller, James C. Shawulu, Mark Arnold, Stefanie Czub, Martin H. Groschup, Anne Balkema-Buschmann

**Affiliations:** 1 Institute of Novel and Emerging Infectious Diseases, Friedrich-Loeffler-Institute, 17493 Greifswald-Insel Riems, Germany; Ivett.Ackermann@fli.de (I.A.); fatolan@yahoo.com (O.I.F.); Markus.Keller@fli.de (M.K.); james.shawulu@ymail.com (J.C.S.); Martin.Groschup@fli.de (M.H.G.); 2 Institute of Veterinary Pathology, Faculty of Veterinary Medicine, Leipzig University, 04103 Leipzig, Germany; reiner.ulrich@vetmed.uni-leipzig.de; 3 Department of Experimental Animal Facilities and Biorisk Management, Friedrich-Loeffler-Institute, 17493 Greifswald-Insel Riems, Germany; Kerstin_Tauscher@gmx.de; 4 Neuroscience Unit, Department of Veterinary Anatomy, Faculty of Veterinary Medicine, University of Ibadan, Ibadan 200284, Nigeria; 5 Department of Veterinary Anatomy, Faculty of Veterinary Medicine, University of Abuja, Abuja 900105, Nigeria; 6 Animal and Plant Health Agency Sutton Bonington, Sutton Bonington, Leicestershire LE12 5RB, UK; Mark.Arnold@apha.gov.uk; 7 Canadian Food Inspection Agency, Lethbridge Laboratory, Lethbridge, AB T1J 3Z4, Canada; stefanie.czub37@gmail.com

**Keywords:** prion protein, BSE, infectivity, PrP^BSE^, cattle, peripheral and central nervous system, protein misfolding cyclic amplification (PMCA)

## Abstract

After oral exposure of cattle with classical bovine spongiform encephalopathy (C-BSE), the infectious agent ascends from the gut to the central nervous system (CNS) primarily via the autonomic nervous system. However, the timeline of this progression has thus far remained widely undetermined. Previous studies were focused on later time points after oral exposure of animals that were already 4 to 6 months old when challenged. In contrast, in this present study, we have orally inoculated 4 to 6 weeks old unweaned calves with high doses of BSE to identify any possible BSE infectivity and/or PrP^BSE^ in peripheral nervous tissues during the first eight months post-inoculation (mpi). For the detection of BSE infectivity, we used a bovine PrP transgenic mouse bioassay, while PrP^BSE^ depositions were analyzed by immunohistochemistry (IHC) and by protein misfolding cyclic amplification (PMCA). We were able to show that as early as 8 mpi the thoracic spinal cord as well as the parasympathetic nodal ganglion of these animals contained PrP^BSE^ and BSE infectivity. This shows that the centripetal prion spread starts early after challenge at least in this age group, which represents an essential piece of information for the risk assessments for food, feed, and pharmaceutical products produced from young calves.

## 1. Introduction

Classical bovine spongiform encephalopathy (C-BSE) is a fatal neurodegenerative prion disease of cattle. Other prion diseases or transmissible spongiform encephalopathies (TSEs) include Creutzfeldt–Jakob disease (CJD) in humans, scrapie in small ruminants, as well as chronic wasting disease (CWD) in cervids. These disorders are caused by the conversion of a host-encoded cellular membrane-bound glycoprotein (PrP^C^) into its abnormal isoform, the pathological prion protein (PrP^TSE^) [1]. BSE is also a zoonotic disease, as the ingestion of BSE-contaminated food has been identified to cause the variant form of CJD [2,3].

In order to minimize the risk of food-borne BSE exposure for humans and animals, specified risk materials (SRM), which may carry BSE infectivity in incubating cattle, were excluded from food, pharmaceuticals, and animal feedstuff. Since the BSE epidemic that had threatened human and animal health for more than two decades has mostly been overcome, BSE regulations have partly been eased. Currently, the SRM list of bovines born in EU member states with negligible BSE risk-as defined in Regulation (EC) No 999/2001 (consolidated version as of 11.2020)-includes the skull (excluding the mandible) with brain and eyes as well as the spinal cord of animals over 12 months of age. For cattle of all ages originating from countries with a controlled or undetermined BSE risk, also the last 4 meters of the small intestine, tonsils, caecum, mesentery, including mesenteric ganglion complex, nerves and fat as well as parts of the vertebral column (including dorsal root ganglia) of animals over 30 months have to be removed and destroyed.

Upon oral prion infection, neuroinvasion into the enteric nervous system (ENS) followed by the CNS and the brain generally seems to follow similar routes in different species. In cattle, the C-BSE agent primarily enters the gut-associated lymphatic tissue (GALT) of the ileum, namely the ileal Peyer’s patch (IPP), in which PrP^BSE^ and BSE infectivity are detectable from early after infection [4,5,6,7,8]. Apart from that, the lymphoreticular system is generally not involved in the BSE pathogenesis in cattle [9,10,11]. In contrast, studies in rodents indicate that agent replication on the cellular membranes of follicular dendritic cells in the GALT may be crucial for prion infection of the enteric nervous system (ENS), subsequent prion spread towards the CNS, and invasion of the brain [12,13,14]. For ovine scrapie, accumulation of the pathological prion protein in the ENS representing the portal to the peripheral nervous system close to the Peyer’s patch (PP) follicles has frequently been described, indicating a contribution of the PPs during neuroinvasion [15,16]. On the other hand, direct neuroinvasion via nerve fibers below the intestinal epithelium (independently from the IPP) has also been discussed for ovine scrapie [15,17]. In an earlier C-BSE study in cattle, indications for both routes were seen, albeit accumulation of PrP^BSE^ in the bovine ENS was shown to occur less frequently than in scrapie-infected sheep [5]. In conclusion, there are three possible roles of the GALT during neuroinvasion: the GALT may either play a crucial key role, or act as an optional intermediary, or it may just be a non-participating bystander of neuronal infection after oral uptake [18].

In an earlier study, BSE infectivity was detectable in the coeliac mesenteric ganglion complex (GMGC) in a high proportion of the animals sacrificed from 16 months post infection (mpi) [19], which functions as the regulator of the digestive tract and contains sympathetic and parasympathetic fibers (mixed ganglion). In cattle, C-BSE prions spread centripetally from the GMGC to the brain mainly via the autonomic nervous system (ANS), primarily via sympathetic structures such as splanchnic nerves, the sympathetic ganglia chain and the cranial cervical ganglion [19,20]. By mouse bioassay, BSE infectivity has been detected in these sympathetic projections, namely from 16 mpi in the splanchnic nerves and the cranial cervical ganglion, and from 24 mpi in the stellate ganglion [19]. Parasympathetic fibers represent alternative routes of BSE spread in cattle, albeit involved to a lesser extent, with detectable infectivity from 20 mpi in the vagal nerve and nodal ganglion. Finally, infectivity was revealed as early as 16 mpi in the thoracic part of the spinal cord [19,20], while PrP^BSE^ accumulations were detectable in its intermediolateral grey column from 24 mpi [19]. Using immunohistochemistry (IHC), PrP^BSE^ has been detected from 44 mpi in the parasympathetic nodal ganglion as well as in the inferior ganglia cells of the thoracic vagal nerve [19]. The latter finding is consistent with the consideration that prions may be in transit rather than actively replicated in nerve fibers [21], which also explains negative IHC results on nerve samples [15,20], such as the lack of PrP^BSE^ detection in the sympathetic splanchnic nerves [19]. The alternative routes of prion centripetal spread towards the brain result in PrP^BSE^ accumulation in the sympathetic cranial cervical ganglion (from 36 mpi positive by IHC), via which the agent may enter the brain at the level of the obex [19]. Besides, spread via parasympathetic routes results in prion accumulation in the dorsal motor nucleus of the vagus (DMNV) as a parasympathetic region of the obex [20]. PrP^BSE^ has been detected by IHC in the obex region as early as 24 mpi [20], while BSE infectivity has been revealed in a sample collected from the caudal medulla of the same animal by mouse bioassay [19]. The sensory trigeminal ganglion was shown to contain PrP^BSE^ from 36 mpi [22,23] and infectivity from 38 mpi [24].

In previous C-BSE pathogenesis studies, cattle were already 4 to 6 months old at the time of challenge [20,25]. In this study, we performed an experimental BSE infection of young, unweaned calves, which has been summarized in a report on the intestinal C-BSE-pathogenesis of these animals [8].

Here, we present data of this early pathogenesis study concerning the early centripetal prion spread towards the brain. For that, we examined peripheral and central nervous tissues of animals sacrificed up to 8 months post-challenge. By providing such detailed analyses of tissues from young calves for the first time, our study fills the gap of data regarding the early C-BSE pathogenesis, especially in young cattle, and provides new knowledge on early prion spread with relevance for risk assessment regarding SRMs and pharmaceuticals.

## 2. Results

In this study, we investigated the early BSE pathogenesis during the first eight months post-inoculation (mpi) in peripheral and central nervous tissues of 18 unweaned calves orally challenged with a classical BSE brain homogenate. To conduct that, we applied detection methods with the highest state-of-the-art sensitivity.

**Figure 1:**
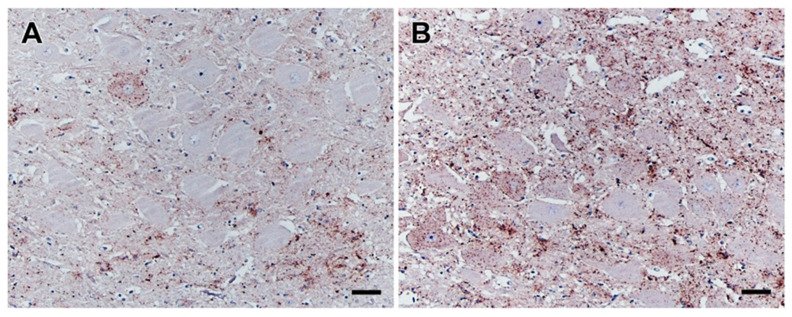
PrP^BSE^ accumulation in the obex of positive control cattle. IHC results obtained for the obex from positive controls IC 01 (36 mpi; (**A**)) and IC 04 (35 mpi; (**B**)); (**A**): a single neuron revealing a intracytoplasmatic and perineuronal staining pattern as well as moderate fine granular PrP^BSE^ accumulation in the neuropil of the dorsal motor nucleus of the vagus nerve (DMNV) of the obex from IC 01 (36 mpi); (**B**): intracytoplasmatic and perineuronal PrP^BSE^ accumulation as well as mild to severe fine to coarse granular accumulation of PrP^BSE^ in the neuropil of the DMNV of the obex from IC 04 (35 mpi); (**A**,**B**): Immunohistochemistry, PrP mAb 6C2, bar 50 µm.

Proof of successful BSE challenge of these 18 preclinical calves was shown by positive ileal Peyer’s patch (IPP) results by IHC and/or PMCA for one animal from the 2 mpi group and all animals from the following groups, while transgenic mouse bioassay revealed BSE infectivity in the IPP samples of all infected animals [8]. In both positive control calves, typical clinical signs of a BSE infection such as hypersensitivity, nervous behavior, and abnormal gait became obvious at 33 mpi (animal IC 04) and 36 months (IC 01), as revealed by the monthly neurological check-up from 24 mpi onwards (Supplementary Table S1). In both cases, a BSE infection was clearly confirmed by IHC (Figure 1) as well as PMCA analysis.

### 2.1. Peripheral Nervous Tissues in Vicinity to the Intestine

For our analysis of the peripheral nervous system (PNS), different approaches were selected for the tissues located in close vicinity to the intestine and tissue located in close vicinity to the central nervous system (CNS).

The vagal and splanchnic nerves as well as the coeliac ganglia of all 18 infected calves that were necropsied in the preclinical stage up to 8 mpi did not contain any detectable PrP^BSE^, as determined by IHC as well as by PMCA. In addition, no BSE infectivity was revealed by Tgbov XV bioassay of those tissues, as all mouse brains available for examination were negative by Western blot (Table 1). The caudal mesenteric ganglion as well as the sympathetic trunk, including paravertebral ganglia of all 18 calves, were negative by IHC. Transgenic mouse bioassay of these samples from the calves of the 6 and 8 mpi groups revealed no BSE infectivity therein.

### 2.2. Peripheral Nervous Tissues in Vicinity to the Brain

**Figure 2:**
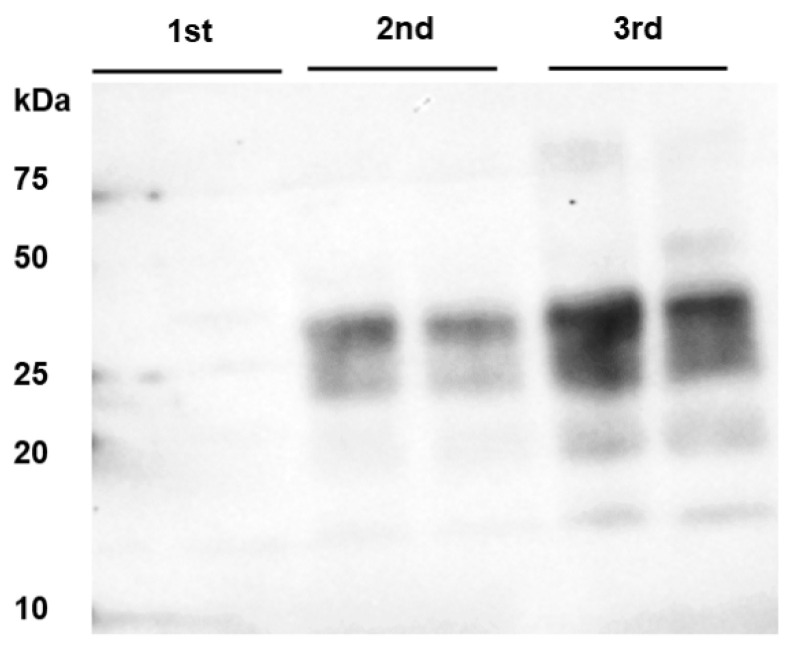
PrP^BSE^ amplification by PMCA in the nodal ganglion at 8 months after challenge. From the third round of PMCA, seeding activity was shown in the nodal ganglion from animal IC 02 (8 mpi); this sample was analyzed in duplicate and subjected to 4 rounds of PMCA; M: marker.

Surprisingly, the sample collected from the nodal ganglion of one of the two animals sacrificed at 8 mpi (IC 02) turned out to contain BSE infectivity, as 2 out of the 34 inoculated mice developed BSE within a mean incubation time of 490 days (SEM 47), as revealed by Western blot analysis of the mouse brain samples (Table 2). Moreover, PrP^BSE^ was detectable in this nodal ganglion sample by PMCA (Figure 2). Based on the mouse bioassay data, the titer of infectivity in the nodal ganglion was estimated to be 0.20 log10 TgBov mouse i.c. LD50/g (95% confidence interval: −0.72–0.91). All other tissues that were sampled in the vicinity of the CNS of this animal and the second animal of this group (cranial cervical ganglion, stellate ganglion, trigeminal ganglion) were negative by IHC, PMCA as well as by transgenic mouse bioassay (Table 2).

Bioassay was performed for the nodal, trigeminal, and cranial cervical ganglia from the six calves sacrificed at 6 mpi, and no BSE infectivity was revealed in any of the inoculated Tgbov XV mouse brains by Western blot analysis (Table 2).

### 2.3. Central Nervous System 

**Figure 3:**
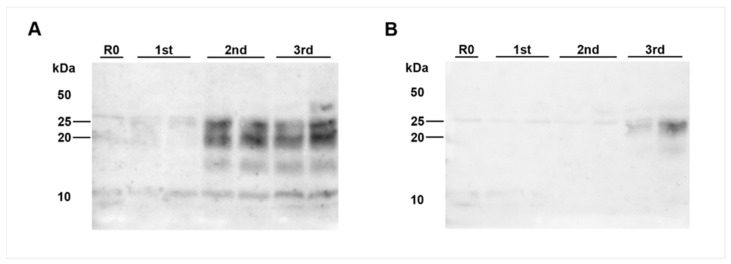
PrP^BSE^ amplification by PMCA in the thoracic spinal cord from a calf at 8 months after challenge. PMCA detected PrP^BSE^ in the thoracic spinal cord segment T7 of IC 02 (8 mpi); (**A**): (+)++ positive PMCA reaction for the homogenate used for inoculation of bioassay mice; (**B**): analyzing a different location of the same T7 sample revealed amplification only in the third round (+ positive reaction); each sample was analyzed in duplicate and subjected to 3 rounds of PMCA. M: marker, R0: analyte homogenate diluted 1:10 in Tgbov XV brain substrate without sonication.

Analysis of the thoracic spinal cord segment T7 collected from IC 02 (8 mpi), that gave a positive result on the nodal ganglion, also revealed BSE infectivity in 8 out of 39 inoculated mice after a mean incubation time of 498 days (SEM 22), as well as seeding activity in all three PMCA rounds (Figure 3A). Based on the mouse bioassay data, the titer of infectivity in the thoracic spinal cord segment T7 was estimated to be 0.81 log10 TgBov mouse i.c. LD50/g (95% confidence interval: 0.31–1.29). It is noteworthy that the same inoculum was used for both analyses. Interestingly, testing a different location within the same tissue sample revealed a positive PMCA result only in the third round of amplification (Figure 3B). The corresponding sample of the other animals of this group (IC 03), as well as all other analyzed CNS samples of both animals (cranial medulla, frontal cortex, cerebellum) remained negative in all tests. The same results were obtained for the six animals sacrificed at 6 mpi (Table 3).

After the detection of PrP^BSE^ by PMCA and BSE infectivity in the thoracic spinal cord of IC 02 (8 mpi), we decided to perform PMCA analyses on the thoracic spinal cord samples obtained during an earlier pathogenesis study [5,20] of preclinical cattle incubating 4 and 8 months after an oral BSE challenge at 4 to 6 months of age (Table S2). PMCA again revealed seeding activity in the thoracic spinal cord of two out of the four available samples from cattle sacrificed at 8 mpi (Supplementary Figure S1).

### 2.4. Positive Controls

Both positive control cattle developed clear clinical signs of BSE after relatively short incubation times of 32 mpi (IC 04) and 36 mpi (IC 01). The animals were then sacrificed at 35 mpi (IC 04) and 36 mpi (IC 01), respectively, and were confirmed as BSE-positive by IHC of the obex region (Figure 1).

**Figure 4:**
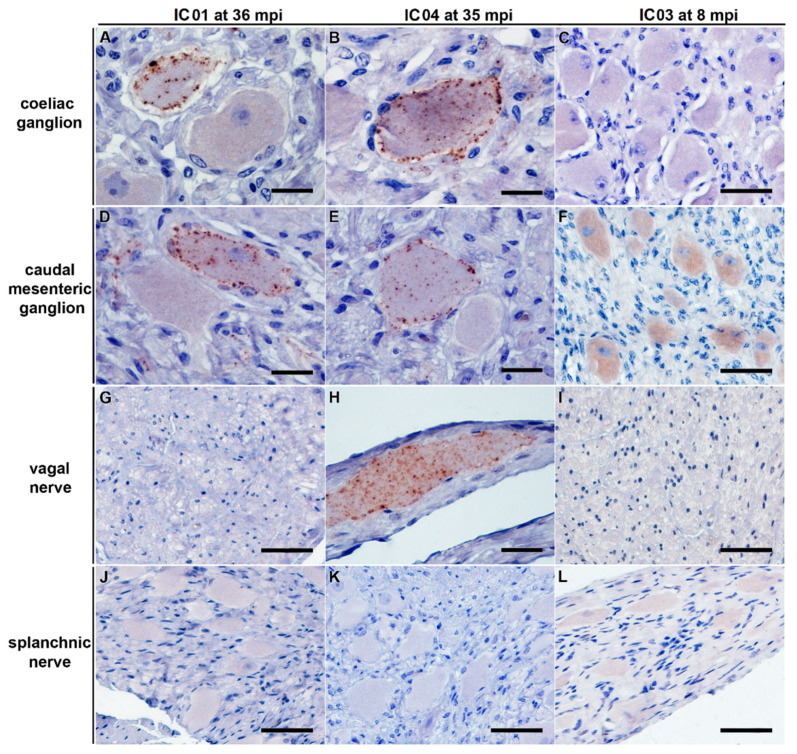
PrP^BSE^ accumulation in the coeliac and caudal mesenteric ganglion as well as the vagal nerve of positive control cattle. IHC results obtained for positive controls IC 01 (36 mpi; (**A**,**D**,**G**,**J**)) and IC 04 (35 mpi; (**B**,**E**,**H**,**K**)) as well as preclinical calf IC 03 (8 mpi; (**C**,**F**,**I**,**L**)); (**A**,**B**,**D**,**E**): fine to coarse granular intracytoplasmatic as well as perineuronal PrP^BSE^ accumulation in a ganglia cell of the coeliac ganglion (**A**,**B**) and the caudal mesenteric ganglion (**D**,**E**) of IC 01 (36 mpi; (**A**,**D**)) and IC 04 (35 mpi; (**B**,**E**)); H: intracytoplasmatic fine to coarse granular accumulation of PrP^BSE^ in a ganglia cell contained in the vagal nerve of IC 04 (35 mpi); (**C**,**F**,**G**,**I**–**L**): no staining reaction in samples from nervous tissues of calf IC 03 (8 mpi; (**C**,**F**,**I**,**L**)) as well as vagal nerve of IC 01 (**G**) and splanchnic nerves of IC 01 (**J**) and 04 (**K**), while the diffuse staining reaction in neural cells in the caudal mesenteric ganglion of IC 03 (**F**) represents an nonspecific background staining; (**A**–**L**): Immunohistochemistry, PrP mAb 6C2; (**A**,**B**,**D**,**E**,**H**): bar 20 µm; (**C**,**F**,**G**,**I**–**L**): bar 50 µm.

In both animals, the coeliac ganglion and caudal mesenteric ganglion samples were detected positive by IHC. This analysis also revealed individual (IC 04; 35 mpi) to few (IC 01; 36 mpi) ganglia cells showing fine to coarse granular intracytoplasmatic as well as perineuronal PrP^BSE^ accumulation (Figure 4A,B,D,E).

**Figure 5:**
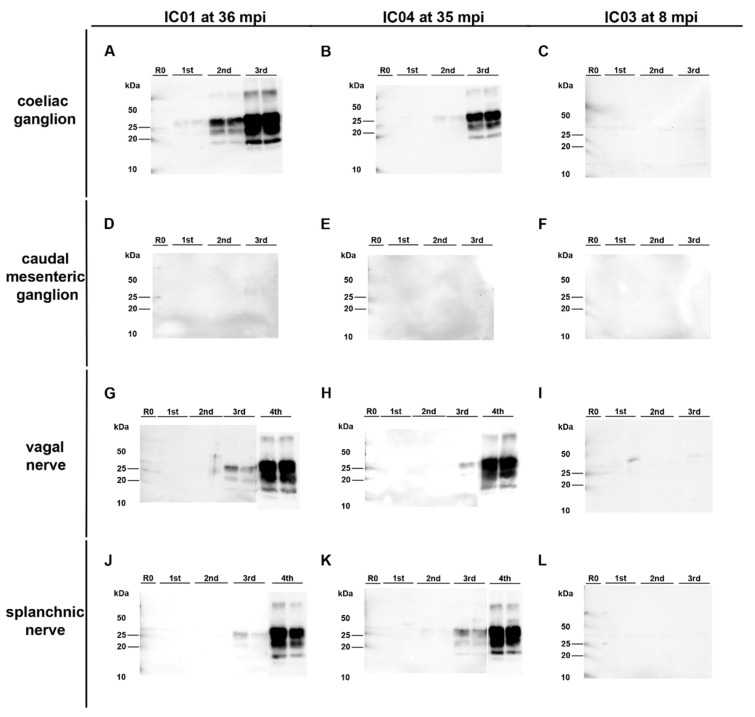
PrP^BSE^ amplification by PMCA in nervous tissue samples from positive control cattle. (**A**,**B**,**D**,**E**): Seeding activity was present in the coeliac ganglion of the positive controls IC 01 (36 mpi; (**A**)) and 04 (35 mpi; (**B**)) as shown by +++ (**A**) and ++ (**B**) positive PMCA reactions, while–in contrast to IHC–no PrP^BSE^ was detectable by PMCA in the caudal mesenteric ganglion of IC 01 (**D**) and 04 (**E**); (**G**,**H**,**J**,**K**): + positive PMCA reactions confirmed the presence of PrP^BSE^ in the parasympathetic vagal nerve (**G**,**H**) and sympathetic splanchnic nerve (**J**,**K**) of IC 01 (**G**,**J**) and 04 (**H**,**K**); (**C**,**F**,**I**,**L**): the mentioned peripheral ganglia and nerves of IC 03 (8 mpi) were negative by PMCA; All analyte tissue samples were analyzed in duplicate and subjected to 3 rounds (1st, 2nd, 3rd) of PMCA. An additional fourth round confirmed PrP^BSE^ amplification of weak positive results obtained with the initial PMCA run of some samples. M: marker, R0: analyte homogenate diluted 1:10 in Tgbov XV brain substrate without sonication.

Surprisingly, PMCA revealed seeding activity in the coeliac ganglia (Figure 5A,D), while it revealed a negative result for the caudal mesenteric ganglia of both animals (Figure 5B,E) (Table 1). Individual ganglia cells in the vagal nerve of IC 04 (35 mpi) displayed an intracytoplasmatic fine to coarse granular staining reaction (Figure 4H), while for IC 01 (36 mpi) the IHC of this nerve remained inconclusive with both mAbs 6C2 and F99. Nevertheless, PMCA confirmed the presence of PrP^BSE^ in the vagal nerve of IC 01 as well as of IC 04 (Figure 5G,H; Table 1). Similarly, the IHC of the splanchnic nerve of both positive control animals remained inconclusive, while PMCA enabled PrP^BSE^ amplification in these samples of IC 01 and 04 (Figure 5J,K; Table 1). PrP^BSE^ was immunohistochemically detectable in the thoracic spinal cord (T7) of both animals (Table 3), while the sympathetic trunk was negative by IHC (Table 2).

Due to ethical reasons and considering the comparably low impact of mouse bioassay results of the tissue samples from the positive control animals, no mouse bioassays were performed on tissues of both positive control cattle.

Meanwhile, analyses of all corresponding samples collected from the negative control animals at 8 mpi gave negative results in all tests (Table S3).

## 3. Discussion

In this study, we aimed at expanding the currently still incomplete data regarding the early C-BSE pathogenesis, especially in young cattle, by analyzing peripheral nerves and ganglia as well as central nervous tissues sampled from calves at particularly short time points between 1 week and 8 months after oral challenge. As recent studies [19,20,22,24] suggested that in calves, neither PrP^BSE^ nor BSE infectivity was expected to be present in nervous tissues, we used the state-of-the-art most sensitive methods (IHC, PMCA, and Tgbov XV mouse bioassay) [5,9,26] to prove or dismiss this hypothesis, while less sensitive methods such as Western Blot and BSE rapid test [5,22,26,27,28] were considered not to be adequate for this task. Moreover, we used a C-BSE-PMCA protocol providing an analytical sensitivity comparable to that of Tgbov XV mouse bioassay, as reported by our recent study, where we were able to show that a 10^−8.3^ dilution of a BSE positive homogenate was detectable by both transgenic mouse bioassay and PMCA [29], and earlier studies [26,30].

We have reported that young unweaned calves were susceptible to an infection with a high BSE dose, as indicated by significant amounts of PrP^BSE^ and high infectivity loads in some of the analyzed ileal Peyer’s patch (IPP) samples from 2 mpi on [8]. However, just one inexplicit indication for a successful neuroinvasion was observed, since PrP^BSE^ was detectable in the enteric nervous system (ENS) of only one animal [8]. Nevertheless, rare PrP^BSE^ accumulation in the ENS cannot be interpreted as proof for a restriction of agent replication to the IPP, as in the current study we were able to show the presence of PrP^BSE^ as well as BSE infectivity from 8 mpi on in peripheral and central nervous tissues.

In this study, we have shown for the first time that upon infection with a high BSE dose that is not likely to be ingested under field conditions, the thoracic spinal cord T7, as a part of the CNS, may contain the BSE agent as early as 8 mpi, as proven by detection of PrP^BSE^ using PMCA, as well as BSE infectivity by mouse bioassay in one of the two animals of this group (IC 02). This finding was unexpected, as infectivity in the spinal cord had thus far only been reported from 16 mpi onwards in our earlier study [19]. Therefore, we also analyzed thoracic spinal cord samples collected during an earlier C-BSE pathogenesis study using older cattle (4 to 6 months at challenge) incubating for 4 and 8 months. Two of these 8 mpi cattle indeed also carried BSE seeding activity (as detected by PMCA) at this location, which supports our results for the calf of this present study sacrificed at 8 mpi. The brainstem was still free of the agent in all animals up to 8 months after infection, as indicated by negative IHC and PMCA results as well as all bioassay results. These brainstem results were in line with our earlier studies showing prion accumulation by IHC and Tgbov XV mouse bioassay in the brainstem starting from 24 mpi [19,20]. Moreover, the parasympathetic nodal ganglion, located close to the brain, was positive for PrP^BSE^ and infectivity at the same levels as reported for the spinal cord sample in the same animal necropsied at 8 mpi (IC 02). This is in contrast to earlier reports where agent detection was described from 20 mpi (mouse bioassay) for the parasympathetic nodal ganglion [19]. As a conclusion, these novel findings that were only possible by the application of several highly sensitive detection methods indicate that a time of up to 8 mpi was sufficient for the agent to spread into peripheral as well as central nervous tissues.

However, we revealed negative results by IHC, PMCA and mouse bioassay for the coeliac and caudal mesenteric ganglion, the splanchnic and vagal nerves from all calves that were sacrificed at these early time points until 8 months after oral challenge. This seems surprising, as earlier studies had shown that prion spread via the coeliac mesenteric ganglion complex (CMGC) and subsequently by the sympathetic splanchnic nerve and the parasympathetic vagal nerve preceded infection of the thoracic spinal cord and the nodal ganglion, respectively [19,20]. In these studies, the earliest detection of BSE infectivity was reported at 16 mpi and that of PrP^BSE^ by IHC at 24 mpi in the CMGC, a mixed ganglion that is involved in the centripetal spread via parasympathetic and sympathetic fibers [19,20]. Infectivity was also present in the sympathetic splanchnic nerves from 16 mpi as well as in the parasympathetic vagal nerve from 28 mpi in the thoracical part and from 20 mpi in its cervical part, respectively [19]. We were able to confirm these pathways for both positive control animals IC 01 and 04 in this study, which showed PrP^BSE^ accumulation in vagal and splanchnic nerves as determined by PMCA. It has been discussed that prions might be in transit without actively replicating in nerves [21], which has been considered as a reason for negative results obtained for nerve samples before [15,20].

It has to be kept in mind that histological and biochemical analyses are based on two different aliquots of one sampling site, which may result in differing results for a weakly positive tissue. This effect may explain the negative PMCA results for the caudal mesenteric ganglion of the positive control animals, while by IHC we were able to detect PrP^BSE^ accumulation in individual ganglia cells of this sample, and this may also be the reason why differing PMCA results were obtained for two locations of the same spinal cord segment from IC 02. Vice versa, PrP^BSE^ amplification by PMCA can complement negative or inconclusive IHC results in samples with low positive results, as suggested by the results for splanchnic and vagal nerves from the positive control animals. This is supported by our earlier study, where ileal Peyer’s patch samples of two infected but preclinical calves were negative or inconclusive by IHC, while a positive PMCA result was in agreement with infectivity detection by mouse bioassay [8].

Finally, our study demonstrates that tissues from infected animals may constitute a BSE exposition risk for humans even if the tissues are derived from young animals. However, it should be considered that for the challenge of the examined cattle, we used a 100 g dose in order to achieve a complete attack rate, while even a dose of 1 mg was shown sufficiently high to cause an infection in cattle in an earlier study [31]. Such a high exposure dose as 100 g of high titer contaminated material is highly unlikely under field conditions [24]. This higher dose will result in a shorter incubation period; based on a previous BSE attack rate study [31], the mean incubation period expected from the inoculum used in the present study would be 42 months, compared to a mean of 66 months estimated for cattle infected in the field in the UK [32]. The impact of a longer incubation period on the timing of infectivity in CNS tissues is uncertain.

The detection of infectivity in the spinal cord in a young animal suggests the possibility that the removal of the skull, including the brain and eyes, as well as the spinal cord at slaughter of bovines over 12 months of age, as defined in Regulation (EC) No 999/2001 (as of May 2017) does not necessarily provide complete consumer protection. However, the likelihood of human exposure to BSE infectivity would also depend on other factors such as the BSE infection prevalence in the cattle population, other control measures in place, and the dose of exposure under field conditions. For countries with a controlled or undetermined BSE risk, risk assessments regarding the public health risk from the spinal cord from cattle under the age of 12 months should be considered, depending on the progress of national eradication campaigns. Moreover, EU legislation provides different regulations for medicinal products, which shall not be, or only in justified exceptions, produced from high-infectivity tissues (category IA based on WHO tables [33]), while the tissues in these three major infectivity categories are grouped regardless of the stage of disease and thus the age of the animals (Note for guidance EMA/410/01 rev.3 [34]). These important regulations regarding consumer protection may be reviewed now, in order to take into account the data of this study.

These novel data also provide indications regarding the age-dependent susceptibility of bovines to BSE infections. We already postulated earlier that an increased prion uptake in combination with a decreased clearance in young calves challenged at 4 to 6 weeks of age (before weaning) favored the agent accumulation in the ileal Peyer’s patch already two months post oral challenge [8]. Neuroinvasion into to the thoracic spinal cord, as shown by PMCA, seems to occur at about 8 mpi in both age groups, challenged at 4 to 6 weeks as well as 4 to 6 months of age. Surprisingly, this is in contrast to a BSE challenge experiment in sheep, which proved the higher BSE susceptibility of young unweaned lambs as compared to older weaned lambs and adults, and also discussed an increased uptake as one reason for this observation [35]. Indeed, the clearing by macrophages in the ileal Peyer’s patch follicles of the young calves was not effective, as indicated by PrP^BSE^ accumulation on follicular dendritic cells (FDCs) as early as 4 mpi [8]. In contrast in the earlier study in older cattle, PrP^BSE^-positive FDCs were only detected from 12 mpi [5]. However, this apparently did not influence neuroinvasion or the speed of prion spread through the nervous system, as illustrated by the presence of PrP^BSE^ at 8 mpi in the thoracic spinal cord of both age groups. The development status of the FDC and of their networks have been shown to differ with age and thereby influence the TSE-susceptibility in mice [36,37] and sheep [38], respectively. Nevertheless, the reportedly reduced agent accumulation and neuroinvasion resulting in decreased disease susceptibility of aged (>600 days old) mice as compared to young (6 to 8 weeks old) mice [36,37] may not apply for the bovines examined in these two studies, as even the animals challenged at 4 to 6 months of age were still young. Otherwise, direct neuroinvasion in the lamina propria mucosae beneath the intestinal epithelium [15,17] may occur independently of FDC status or development of FDC networks and may, therefore, provide another explanation for our observation. The accumulation of PrP^BSE^ in a submucosal plexus of the ENS from one calf at two mpi in the absence of detectable PrP^BSE^ in the ileal Peyer’s patch follicles may reflect this direct neuroinvasion [8]. Finally, a third explanation should consider that replication on FDCs in other lymphoreticular organs may play a role for the further progression of prion infection as reported in sheep and mice [14,39,40]. Moreover, the acceleration of disease progression may not occur in bovines as the lymphoreticular system, except for the IPP, is rarely involved in cattle BSE pathogenesis [9,10,11].

## 4. Materials and Methods

### 4.1. Animals

A total of 20 unweaned Simmental calves aged 4–6 weeks were orally challenged with classical BSE using a brainstem homogenate of clinically diseased cattle, as described before [8]. The infected animals were designated as IC (infected calf) and the uninfected animals as CC (control calf). The inoculum had an LD_50_-titer of 10^−5.730^ (95% confidence interval, 10^−6.569^–10^−4.891^) [8] as experimentally determined by titration in Tgbov XV mice [9]. A total of 18 infected calves were euthanized and necropsied at predetermined time points of 1 week (2 animals) as well as 2 (2 animals), 4, 6 (6 animals each), and 8 (2 animals) months post-inoculation (mpi). At necropsy, tissue samples from the central and peripheral nervous system, the lymphoreticular system, and the gastrointestinal tract were collected under TSE-sterile conditions. As positive controls, 2 infected animals were kept to monitor the development of clinical signs of BSE, and were examined by a monthly neurological check-up starting from 24 mpi [8]. Additionally, 2 calves were inoculated with a BSE-negative brainstem homogenate and served as negative controls that were sacrificed at 8 mpi (Table S3).

### 4.2. Tissue Samples

During necropsy, tissue samples were cut in halves for formalin-fixed tissue for the IHC analyses and frozen material for the protein misfolding cyclic amplification (PMCA) and bioassay studies. Samples of the central nervous system (CNS) and peripheral nervous system (PNS) were examined from the 20 BSE-challenged animals and the 2 negative controls (*n* = 22). The current state of knowledge led to the working hypothesis that nervous tissues of the PNS and CNS were most probably free of PrP^BSE^ and BSE prion infectivity up to 8 mpi.

Therefore, cerebellum, frontal cortex, and peripheral nervous tissues located close to the brain (stellate, nodal, trigeminal, and cranial cervical ganglia), were analyzed by the highly sensitive mouse bioassay and PMCA in order to be able to detect even trace amounts if present in these samples. Transgenic Tgbov XV mouse bioassays were also performed on selected samples collected from the calves of the 4 and 8 mpi groups in order to keep the bioassay mice numbers at a minimum. Peripheral tissue samples in the vicinity of the intestine (coeliac and caudal mesenteric ganglia, splanchnic nerve, vagal nerve, and sympathetic trunk as well as the CNS samples of the brainstem and the thoracic spinal cord segment T7) where positive results were anticipated by the beginning of the study were not only examined by mouse bioassay and PMCA, but also by immunohistochemistry (IHC) to evaluate the involved cellular compartments of any PrP^BSE^ accumulation that may be traceable in those samples.

### 4.3. Comparative Analysis of Samples from Cattle Challenged 4–6 Months of Age

In order to be able to compare some unexpected results obtained for samples of the thoracic spinal cord and the nodal ganglion from calves sacrificed at 4 and 8 mpi, the corresponding samples that had been collected at 4 and 8 mpi during a BSE pathogenesis study performed in cattle that were challenged at the age of 4 to 6 months [5,20] were also analyzed by PMCA.

### 4.4. Immunohistochemistry (IHC) and Histopathological Examination

Tissue samples were processed and immunohistochemical staining was performed as described before [8]. Briefly, samples were fixed in 4% neutral buffered formaldehyde for at least 2 weeks. Fixed tissues were cut into blocks, dehydrated, and embedded in paraffin wax according to standard histopathological methods. Sections (3 µm) were prepared and mounted on SuperFrost Plus slides (Thermo Fisher Scientific Gerhard Menzel, Braunschweig, Germany). For samples of the 18 challenged calves between 1 week and 8 months post-inoculation (*n* = 18) and negative controls (*n* = 2), a serial section procedure [5] was used to examine 5 different levels per block with a plane distance of about 30 µm and to obtain a depth of approximately 195 µm per block. In contrast, tissue samples from the 2 positive control animals (IC 01 and 04, *n* = 2) where positive results were expected, only 1 level per block was analyzed first by IHC, and only in the case of a negative result, additional levels were examined. For the histopathological examination, hematoxylin-eosin staining was performed according to standard histological methods.

With modifications depending on the type of tissues, IHC staining was performed as described before [8]. Two PrP-specific monoclonal antibodies (mAbs) were used: routine IHC staining was performed using mAb 6C2 (Wageningen Bioveterinary Research, Lelystad, The Netherlands), whereas additional sections were stained with mAb F99 (F99/97.6.1, VMRD, Pullman, WA, USA) for verification of inconclusive results. As a negative control antibody, on corresponding sections, the anti-PrP mAb 3F4 (CHEMICON International, Temecula, WA, USA) was applied, which does not bind to bovine PrP [41]. After rehydration, sections were pretreated with 98% formic acid for 15 min and rinsed in tap water for 5 min. Endogenous peroxidase was blocked with 3% H_2_O_2_ in distilled water for 30 min. Sections underwent hydrated autoclaving in citrate buffer at 121 °C for 20 min. As the central nervous system is rich in endogenous biotin, for samples of obex and spinal cord segment T7, inhibition of endogenous biotin was accomplished using an avidin/biotin blocking kit (Vector Laboratories, Burlingame, CA, USA) as described before [8]. All slides were analyzed for PrP^BSE^ depositions by light microscopy.

### 4.5. Tgbov XV Mouse Bioassay

Selected samples were examined by bioassay, using transgenic mice over-expressing bovine PrP [9]. Depending on the probability that the tissue might carry BSE infectivity, 20 or 40 Tgbov XV mice per group were intracerebrally inoculated using 30 µL of a 10% tissue homogenate diluted in sterile 0.9% saline solution. In cases where the quantity of tissue samples was too low to prepare a 10% homogenate, a 2% tissue homogenate had to be used instead.

Tissues in the vicinity to the intestine, precisely the coeliac ganglia, splanchnic and vagal nerves, from the infected calves sacrificed between 1 week and 8 months post-challenge (*n* = 18) and negative controls (*n* = 2) were analyzed by inoculating 20 Tgbov XV mice per group in the transgenic mouse bioassay. In addition, mouse bioassays with 40 mice per group were performed for selected samples from the calves of the 6 and 8 mpi groups (*n* = 10). These tissue samples included the caudal mesenteric ganglia, the central nervous system (thoracic spinal cord segment T7, cranial medulla, and cerebellum), and peripheral tissues nearby the brain (sympathetic trunk, nodal, trigeminal, and cranial cervical ganglia).

All mice were monitored for the onset of clinical signs at least twice per week. Animals showing at least 2 clinical signs indicative of a BSE infection, such as hind limb paresis, abnormal tail tonus, behavioral changes and weight loss over several consecutive days [9] were sacrificed and brain samples were collected. The brains were analyzed for the presence of PrP^BSE^ by precipitation using phosphotungstic acid (PTA) followed by digestion with 50 µg/mL Proteinase K at 55 °C for 1 h and Western blot using mAb L42 at a concentration of 0.4 μg/mL (r-biopharm, Darmstadt, Germany) as detection antibody [42]. Results of mice incubating at least 100 dpi were taken into evaluation.

### 4.6. Protein Misfolding Cyclic Amplification (PMCA)

The earlier described PMCA protocol [26,30] was applied with some modifications as described before [8,29]. Briefly, brain tissue from Tgbov XV transgenic mice [43] that were collected after perfusion of the mice with PBS containing 5 mmol/l EDTA and immediately frozen in liquid nitrogen was used as the PrPC source for the PMCA reaction. Brain samples were homogenized to a concentration of 10% (*w*/*v*) in PMCA conversion buffer to prepare the substrate solution. Analyte tissue samples were homogenized at 10% (*w*/*v*) in 0.9% saline solution. 10 µL aliquots of the analyte homogenates were suspended in 90 µL Tgbov XV brain substrate and transferred into 0.5 mL reaction tubes. The template for the positive control PMCA reaction was a 10% (*w*/*v*) homogenate of bovine brain tissue in PBS of which serial dilutions (10^−3^, 10^−6^, 10^−9^) were prepared in substrate solution. A brain sample of a confirmed BSE-negative cattle served as a negative control.

Samples were routinely subjected to 3 rounds of PMCA with every 48 cycles of sonication for 20 s a time at a potency of 210–250 W (level 8), followed by a 30 min incubation. For samples that were close to the PMCA detection limit, a fourth round was performed in addition, including the positive and negative control samples. The experiment was considered valid if at least the 10^−3^ and 10^−6^ dilutions were clearly identified as positive, and the negative control gave a negative result.

The obtained results were interpreted as follows: a clear PrP^BSE^ signal in all 3 PMCA rounds was interpreted as +++ positive, a signal in the second and third PMCA round was interpreted as ++ positive, and a signal only in the third PMCA round was interpreted as + positive. For samples where a fourth round was performed, the results were reported as (+) if only the fourth round gave a positive result.

### 4.7. Estimation of the Titer of Infectivity

The titer of infectivity in each sample was estimated using the method developed by Arnold et al. [24]. It used both the attack rate and the observed incubation periods to estimate titer and thus was able to provide a more accurate estimation of titer than using either attack rate or incubation period data alone. The relationship between the titer of inoculum and the probability of infection, and the length of the incubation period was derived from data from 3 separate endpoint titrations in cattle [8,9,20]. Normal distribution for the relationship between dose and incubation period was assumed, and the probability of infection versus dose was assumed to follow a logistic regression curve (Figure S2).

## 5. Conclusions

In summary, we detected PrP^BSE^ and BSE infectivity as early as 8 mpi in the nodal ganglion as well as in the thoracic spinal cord from one calf challenged before weaning in this study and also at eight mpi in the thoracic spinal cord sampled from cattle challenged at 4 to 6 months of age during an earlier pathogenesis study [5,20]. This current study considerably expands the existing data on the early C-BSE pathogenesis by demonstrating that after challenge with an unnaturally high dose of 100 g BSE-positive brainstem tissue, parts of the peripheral and central nervous system from cattle may already contain PrP^BSE^ and BSE infectivity after short time periods up to 8 months after oral infection, which should be considered relevant information for risk assessments for food and pharmaceutical products.

## Figures and Tables

**Table 1:** Results for peripheral nervous tissues in the vicinity to the intestine were obtained by IHC, PMCA, and Tgbov XV mouse bioassay.

Time Point Post Inocu-lation	Animal ID	Ganglion Coeliacum			Ganglion Mesenteriale Caudale			Nervus Splanchnicus Major			Truncus Sympathicus (incl. Paravertebral Ganglia)			Nervus Vagus (Thoracic Part)		
		IHC	PMCA	BA	IHC	PMCA	BA	IHC	PMCA	BA	IHC	PMCA	BA	IHC	PMCA	BA
1 week	IC 17	neg.	neg.	0/20 >732	neg. †	neg.	n. d.	neg.	neg.	0/20 >733	neg.	neg.	n. d.	neg.	neg.	0/20 >734
	IC 18	neg.	neg.	0/20 >731	neg.	neg.	n. d.	neg.	neg.	0/20 >731	neg.	neg.	n. d.	neg.	neg.	0/19 >734
2 m	IC 15	neg.	neg.	0/9 >734	neg.	neg.	n. d.	neg.	neg.	0/20 >733	neg.	neg.	n. d.	neg.	neg.	0/20 >731
	IC 16	neg.	neg.	0/18 >734	neg.	neg.	n. d.	neg.	neg.	0/20 >733	neg.	neg.	n. d.	neg.	neg.	0/20 >732
4 m	IC 11	neg.	neg.	0/20 >734	neg. †	neg.	n. d.	neg.	neg.	0/22 >733	neg.	neg.	n. d.	neg.	neg.	0/20 >731
	IC 12	neg.	neg.	0/20 >743	neg.	neg.	n. d.	neg.	neg.	0/19 >743	neg.	neg.	n. d.	neg.	neg.	0/20 >740
	IC 13	neg.	neg.	0/20 >731	neg.	neg.	n. d.	neg.	neg.	0/20 >731	neg.	neg.	n. d.	neg.	neg.	0/20 >731
	IC 14	neg.	neg.	0/20 >693	neg.	neg.	n. d.	neg.	neg.	0/21 >764	neg.	neg.	n. d.	neg.	neg.	0/20 >731
	IC 19	neg.	neg.	0/20 >734	neg.	neg.	n. d.	neg.	neg.	0/20 >731	neg.	neg.	n. d.	neg.	neg.	0/20 >731
	IC 20	neg.	neg.	0/19 >750	neg.	neg.	n. d.	neg.	neg.	0/20 >741	neg.	neg.	n. d.	neg.	neg.	0/20 >745
6 m	IC 05	neg.	neg.	0/20 >732	neg. †	neg.	0/39 >726	neg.	neg.	0/20 >732	neg.	neg.	0/40 >728	neg.	neg.	0/19 >732
	IC 06	neg.	neg.	0/19 >743	neg.	neg. #	0/40 >733	neg.	neg.	0/19 >750	neg.	neg.	0/40 >732	neg.	neg.	0/20 >733
	IC 07	neg.	neg.	0/20 >732	neg.	neg.	0/38 >734	neg.	neg.	0/20 >732	neg.	neg.	0/40 >731	neg.	neg.	0/20 >735
	IC 08	neg.	neg.	0/20 >733	neg.	neg.	0/38 >754	neg.	neg.	0/19 >734	neg.	neg.	0/40 >740	neg.	neg.	0/18 >730
	IC 09	neg.	neg.	0/20 >734	neg.	neg.	0/39 >728	neg.	neg.	0/20 >734	neg.	neg.	0/39 >731	neg.	neg.	0/20 >732
	IC 10	neg.	neg.	0/20 >731	neg.	neg. #	0/40 >733	neg.	neg.	0/20 >732	neg.	neg.	0/39 >731	neg.	neg.	0/20 >731
8 m	IC 02	neg.	neg.	0/20 >734	neg.	neg.	0/40 >734	neg.	neg.	0/20 >730	neg.	neg.	0/43 >731	neg.	neg.	0/20 >734
	IC 03	neg.	neg.	0/20 >734	neg.	neg.	0/39 >737	neg.	neg.	0/19 >764	neg.	neg.	0/38 >728	neg.	neg.	0/20 >733
35 m	IC 04	+ *	++	n. d.	(+) *	neg.	n. d.	inc.	+	n. d.	neg.	neg.	n. d.	+	(+)	n. d.
36 m	IC 01	+ *	+	n. d.	(+) *	neg.	n. d.	inc.	(+)	n. d.	neg.	neg.	n. d.	inc.	(+)	n. d.

IHC: immunohistochemistry, neg.: negative, inc.: inconclusive, m: months, *: in one level of the block; †: no ganglia cells, but only nerve fibers were available for analyses; PMCA: protein misfolding cyclic amplification, +: positive in the third round, ++: positive from the second round; neg.: negative, n. d.: not done, #: a 2% homogenate was analyzed due to tissue size; BA: bioassay in Tgbov XV mice: positive/inoculated mice and mean incubation time in days ± standard error of the mean (SEM) or survival time of oldest negative mice.

**Table 2:** Results for peripheral nervous tissues closer to the brain were achieved by PMCA and Tgbov XV mouse bioassay.

Time Point Post Inoculation	Animal ID	Ganglion Cervicale Craniale		Ganglion Stellatum		Ganglion Nodosum		Ganglion Trigeminale	
		PMCA	BA	PMCA	BA	PMCA	BA	PMCA	BA
1 week	IC 17	neg.	n. d.	neg.	n. d.	neg.	n. d.	neg.	n. d.
	IC 18	neg.	n. d.	neg.	n. d.	neg.	n. d.	neg.	n. d.
2 m	IC 15	neg.	n. d.	neg.	n. d.	neg.	n. d.	neg.	n. d.
	IC 16	neg.	n. d.	neg.	n. d.	neg.	n. d.	neg.	n. d.
4 m	IC 11	neg.	n. d.	neg.	n. d.	neg.	n. d.	neg.	n. d.
	IC 12	neg.	n. d.	neg.	n. d.	neg.	n. d.	neg.	n. d.
	IC 13	neg.	n. d.	neg.	n. d.	neg.	n. d.	neg.	n. d.
	IC 14	neg.	n. d.	neg.	n. d.	neg.	n. d.	neg.	n. d.
	IC 19	neg.	n. d.	neg.	n. d.	neg.	n. d.	neg.	n. d.
	IC 20	neg.	n. d.	neg.	n. d.	neg.	n. d.	neg.	n. d.
6 m	IC 05	neg.	0/40, >731	neg.	n. d.	neg.	0/39, >735	neg.	0/39, >730
	IC 06	neg.	0/38, >731	neg.	n. d.	neg.	0/40, >731	neg.	0/39, >730
	IC 07	neg.	0/39, >731	neg.	n. d.	neg.	0/39, >732	neg.	0/39, 730
	IC 08	neg.	0/40, >743	neg.	n. d.	neg.	0/30, >757	neg.	0/39, >731
	IC 09	neg.	0/39, >731	neg.	n. d.	neg. #	0/39, >733	neg.	0/40, >731
	IC 10	neg. #	0/38, >733	neg.	n. d.	neg.	0/38, >732	neg.	0/39, >731
8 m	IC 02	neg.	0/39, >736	neg.	n. d.	+	2/34, 490 ± 66 (47)	neg.	0/40, >733
	IC 03	neg.	0/40, >731	neg.	n. d.	neg.	0/39, >732	neg.	0/40, >735
35 m	IC 04	neg	n. d.	++	n. d.	neg	n. d.	+++	n. d.
36 m	IC 01	neg	n. d.	+	n. d.	neg	n. d.	+	n. d.

PMCA: protein misfolding cyclic amplification, +: positive in the third round, ++: positive from the second round, +++: positive from the first round; neg.: negative, m: months, n. d.: not done; # a 2% homogenate was analyzed due to tissue size; BA: bioassay in Tgbov XV mice: positive/inoculated mice and mean incubation time in days ± standard error of the mean (SEM) or survival time of oldest negative mice.

**Table 3:** Results obtained for samples of the central nervous system together with the clinical status of all challenged calves.

Time Point Post Inoculation	Animal ID	Infection Status	Obex	Cranial Medulla		Frontal Cortex		Cerebellum		Thoracic Spinal Cord T7		
			IHC	PMCA	BA	PMCA	BA	PMCA	BA	IHC	PMCA	BA
1 week	IC 17	Preclinical	neg.	neg.	n. d.	neg.	n. d.	neg.	n. d.	neg.	neg.	n. d.
	IC 18	Preclinical	neg.	neg.	n. d.	neg.	n. d.	neg.	n. d.	neg.	neg.	n. d.
2 m	IC 15	Preclinical	neg.	neg.	n. d.	neg.	n. d.	neg.	n. d.	neg.	neg.	n. d.
	IC 16	Preclinical	neg.	neg.	n. d.	neg.	n. d.	neg.	n. d.	neg.	neg.	n. d.
4 m	IC 11	Preclinical	neg.	neg.	n. d.	neg.	n. d.	neg.	n. d.	neg.	neg.	n. d.
	IC 12	Preclinical	neg.	neg.	n. d.	neg.	n. d.	neg.	n. d.	neg.	neg.	n. d.
	IC 13	Preclinical	neg.	neg.	n. d.	neg.	n. d.	neg.	n. d.	neg.	neg.	n. d.
	IC 14	Preclinical	neg.	neg.	n. d.	neg.	n. d.	neg.	n. d.	neg.	neg.	n. d.
	IC 19	Preclinical	neg.	neg.	n. d.	neg.	n. d.	neg.	n. d.	neg.	neg.	n. d.
	IC 20	Preclinical	neg.	neg.	n. d.	neg.	n. d.	neg.	n. d.	neg.	neg.	n. d.
6 m	IC 05	Preclinical	neg.	neg.	0/40, >735	neg.	n. d.	neg.	0/39, >732	neg.	neg.	0/40; >732
	IC 06	Preclinical	neg.	neg.	0/40, >735	neg.	n. d.	neg.	0/43, >735	neg.	neg.	0/37; >732
	IC 07	Preclinical	neg.	neg.	0/40, >733	neg.	n. d.	neg.	0/40, >732	neg.	neg.	0/38; >735
	IC 08	Preclinical	neg.	neg.	0/38, >735	neg.	n. d.	neg.	0/39, >748	neg.	neg.	0/39; >731
	IC 09	Preclinical	neg.	neg.	0/40, >735	neg.	n. d.	neg.	0/36, >732	neg.	neg.	0/40, >732
	IC 10	Preclinical	neg.	neg.	0/39, >735	neg.	n. d.	neg.	0/41, >731	neg.	neg.	0/39, >732
8 m	IC 02	Preclinical	neg.	neg.	0/38, >733	neg.	n. d.	neg.	0/37, >733	neg.	++	8/39, 498 ± 24 (22)
	IC 03	Preclinical	neg.	neg.	0/40, >735	neg.	n. d.	neg.	0/38, >735	neg.	neg.	0/39, >732
35 m	IC 04	pos.control	+	+++	n. d.	+	n. d.	+++	n. d.	+(+)	+++	n. d.

36 m	IC 01	pos. control	++	+++	n. d.	+++	n. d.	+++	n. d.	+++	++	n. d.


IHC: immunohistochemistry, neg.: negative, pos.: positive, m: months, PMCA: protein misfolding cyclic amplification, +: positive in the third round, ++: positive from the second round, +++: positive from the first round; neg.: negative, n. d.: not done; BA: bioassay in Tgbov XV mice: positive/inoculated mice and mean incubation time in days ± standard error of the mean (SEM) or survival time of oldest negative mice.

## Data Availability

The data presented in this study are available on request from the corresponding author.

## Supplementary Materials

The following are available online at https://www.mdpi.com/article/10.3390/ijms222111310/s1.
