# Supplementary material for: Prion Infectivity and PrPBSE in the Peripheral and Central Nervous System of Cattle 8 Months Post Oral BSE Challenge
Source: Int J Mol Sci. 2021 Oct 20;22(21):11310. doi: 10.3390/ijms222111310 (PMC8583047; doi:10.3390/ijms222111310)
Supplement: Supplementary file 1 [file ijms-22-11310-s001.zip › Table S1 new.pdf]

Animal ID	Frontal Approach				Blink Reflex				Face Sensitivity				Flashlight Test				Frontal Movement				Broom Test				Clap Test		
level	1	2	3	4	1	2	3	4	1	2	3	4	1	2	3	4	1	2	3	4	1	2	3	4	1	2	3
IC 04	x				x					x	x		x	x				x	x		x					x	x
IC 01	x				x				x	x			x	x			x	x				x			x		

Animal ID	Gait				Behavior				Obstacle (Bar)				Body Condition					Total score
level	1	2	3	4	1	2	3	4	1	2	3	4	1	2	3	4	5	min. 11 – max. 41
IC 04		x					x	x			x				x	x		23
IC 01		x			x	x			x					x				16
