# Supplementary material for: Prion Infectivity and PrPBSE in the Peripheral and Central Nervous System of Cattle 8 Months Post Oral BSE Challenge
Source: Int J Mol Sci. 2021 Oct 20;22(21):11310. doi: 10.3390/ijms222111310 (PMC8583047; doi:10.3390/ijms222111310)
Supplement: Supplementary file 1 [file ijms-22-11310-s001.zip › Table S2.pdf]

Time point post infection	Animal ID	Thoracic spinal cord
		PMCA
4 months (n = 2)	IT 19	neg.
	IT 45	neg.
8 months (n = 2)	IT 14	neg.
	IT 20	++
	IT 39	++
	IT 55	neg.
