# Supplementary material for: Prion Infectivity and PrPBSE in the Peripheral and Central Nervous System of Cattle 8 Months Post Oral BSE Challenge
Source: Int J Mol Sci. 2021 Oct 20;22(21):11310. doi: 10.3390/ijms222111310 (PMC8583047; doi:10.3390/ijms222111310)
Supplement: Supplementary file 1 [file ijms-22-11310-s001.zip › Table S3 new.pdf]

Tissue sample	CC 01 (8 mpi)			CC 02 (8 mpi)		
	IHC	PMCA	BA	IHC	PMCA	BA
**Obex**	neg.	n. d.	n. d.	neg.	n. d.	n. d.
**Cranial medulla**	n. d.	neg.	0/20, > 730	n. d.	neg.	0/20, > 730
**Ganglion coeliacum**	neg.	neg.	0/20, > 732	neg.	neg.	0/20, > 729
**Ganglion mesenteriale caudale**	neg.	neg.	n. d.	neg.	neg.	n. d.
**Nervus splanchnicus major**	neg.	neg.	0/20, > 732	neg.	neg.	0/20, > 741
**Truncus sympathicus (incl. paravertebral ganglia)**	neg.	o. a.	n. d.	neg.	o. a.	n. d.
**Nervus vagus (thoracic part)**	neg.	neg.	0/20, > 733	neg.	neg.	0/19, > 733
**Thoracic spinal cord T7**	neg.	neg.	0/20, > 740	neg.	o. a.	0/19, > 740
**Frontal cortex**	n. d.	o. a.	n. d.	n. d.	o. a.	n. d.
**Cerebellum**	n. d.	o. a.	0/19, > 753	n. d.	o. a.	0/19, > 745
**Ganglion cervicale craniale**	n. d.	o. a.	n. d.	n. d.	o. a.	n. d.
**Ganglion stellatum**	n. d.	o. a.	n. d.	n. d.	o. a.	n. d.
**Ganglion nodosum**	n. d.	o. a.	0/20, > 741	n. d.	o. a.	0/20, > 740
**Ganglion trigeminale**	n. d.	o. a.	0/20, > 742	n. d.	o. a.	0/19, > 740
