# Supplementary figures and images for: Prion Infectivity and PrPBSE in the Peripheral and Central Nervous System of Cattle 8 Months Post Oral BSE Challenge
Source: Int J Mol Sci. 2021 Oct 20;22(21):11310. doi: 10.3390/ijms222111310 (PMC8583047; doi:10.3390/ijms222111310)

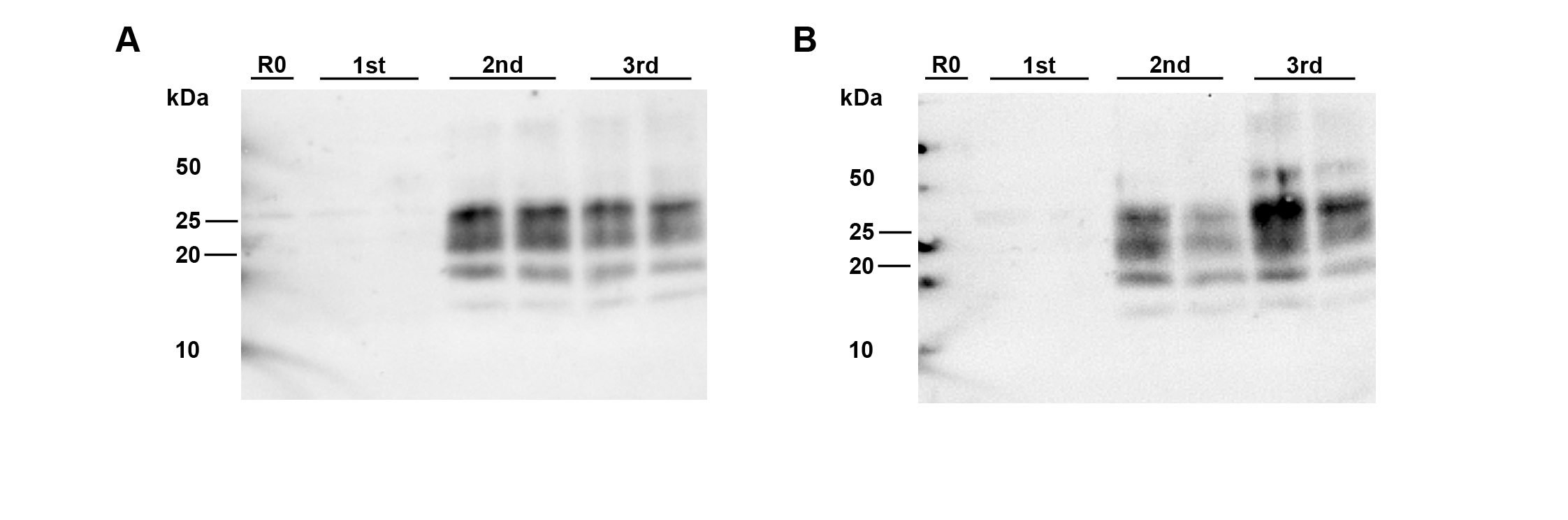

Supplement: Supplementary file 1 [file ijms-22-11310-s001.zip › Figure S1.tif]

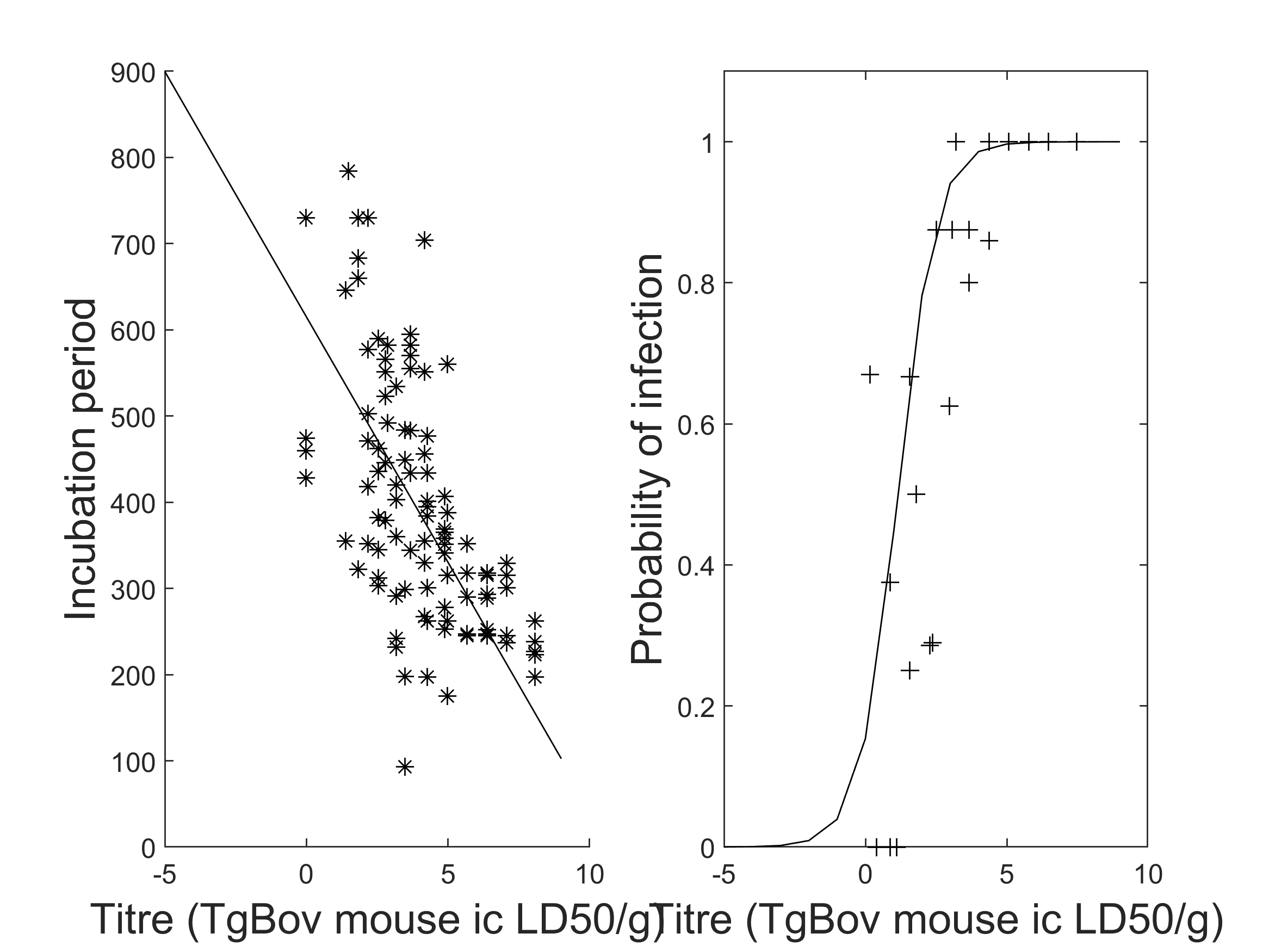

Supplement: Supplementary file 1 [file ijms-22-11310-s001.zip › Figure S2.tif]
